# The Impact of Exploitative Leadership and Moral Disengagement on Nurses’ Work Withdrawal Behaviors: A Mediation Model Using the Partial Least Squares Approach

**DOI:** 10.1155/jonm/6932401

**Published:** 2025-12-31

**Authors:** Mennat Allah G. Abou Zeid, Amirat A. Al-Sabeely, Ibrahim Abdullatif Ibrahim

**Affiliations:** ^1^ Nursing Administration and Education Department, College of Nursing, Prince Sattam Bin Abdulaziz University, Al-Kharj, Saudi Arabia, psau.edu.sa; ^2^ Department of Nursing Sciences, College of Applied Medical Sciences, Shaqra University, Shaqra, Saudi Arabia, su.edu.sa; ^3^ Department of Nursing Administration, Faculty of Nursing, Mansoura University, Mansoura, Egypt, mans.edu.eg

**Keywords:** leadership, moral disengagement, nursing staff, occupational stress, workplace behavior

## Abstract

**Background:**

Work withdrawal behaviors threaten patients’ safety and care quality. While exploitative leadership is a potential contributor to these negative behaviors, its impact remains underexplored in nursing context. Additionally, the mediating mechanism of moral disengagement in this association is understudied.

**Aim:**

This research aimed to investigate the mediating function of moral disengagement in the linkage between exploitative leadership and work withdrawal behaviors, framed by social cognitive and social exchange theories.

**Methods:**

This quantitative, cross‐sectional study included a convenience sample of 223 nurses recruited from two healthcare settings. Data were collected between February and April 2025 using an online survey comprising three validated and reliable scales in addition to demographic questionnaire. The proposed hypotheses were tested using the partial least square approach.

**Results:**

Exploitative leadership demonstrated a significant positive direct effect on both moral disengagement (*β* = 0.616, *t* = 17.798, *p* < 0.001) and work withdrawal behaviors (*β* = 0.421, *t* = 5.592, *p* < 0.001). Furthermore, moral disengagement had a significant positive effect on work withdrawal behaviors (*β* = 0.196, *t* = 2.365, *p* < 0.05). The indirect effect of exploitative leadership on work withdrawal behaviors, mediated by moral disengagement, was also significant (*β* = 0.121, *t* = 2.238, *p* < 0.05).

**Conclusion:**

This research underscores the critical role of moral disengagement in elucidating how exploitative leadership fosters nurses’ job withdrawal behaviors. These results underscore the need for healthcare organizations to advocate for ethical leadership practices and address processes that compromise nurses’ moral involvement to enhance nurse retention and ensure high‐quality patient care.

## 1. Introduction

Leadership is very important in establishing healthcare settings since it has a direct effect on both nurse well‐being and patient outcomes [1, 2]. Effective leadership is recognized for improving nurse engagement and retention, while destructive leadership, such as exploitative leadership, may lead to disengagement and withdrawal behaviors [3–6].

Exploitative leadership may undermine employee morale, erode trust, and induce emotional and psychological distress [7, 8]. Studies indicate that exploitative leadership might promote job withdrawal behaviors [9–11]. In health care organizations, work withdrawal behaviors had a serious threat to patient safety, health care quality, and organizational suitability [12, 13].

Despite evidence linking exploitative leadership to withdrawal behaviors [9], there is limited empirical focus on these dynamics within the nursing profession, and even fewer studies have explored the mediating effect of moral disengagement in the Saudi nursing context. Addressing these gaps offers important implications for leadership development, organizational ethics, and interventions to reduce withdrawal behaviors in healthcare settings.

## 2. Background

### 2.1. Exploitative Leadership

Exploitative leadership is a destructive leadership style characterized by self‐serving actions that put the leader’s interests above those of subordinates and organizational objectives [14]. Exploitative leadership undermines trust, fosters perceptions of injustice and job insecurity, and contributes to a toxic organizational climate, increasing the likelihood that employees will withhold knowledge and engage in counterproductive behaviors [15–17]. In health care settings, particularly in nursing, exploitative leadership poses significant risks to both staff well‐being and patient outcomes. Nurses exposed to such leadership often report elevated psychological distress, job dissatisfaction, dimmish quality of care, greater absenteeism, and stronger intention to leave the profession [7, 18, 19].

### 2.2. Work Withdrawal Behaviors

Work withdrawal behaviors represent a continuum of cognitive, emotional, and physical actions through which employees distance themselves from their work roles. Wise defines work withdrawal as actions that “permit employees to withdraw from work participation,” including absenteeism, psychological disengagement, reduced effort, and turnover intentions [20]. Within nursing, however, withdrawal behaviors often manifest in discipline‐specific ways that directly affect patient care processes. Empirical studies have shown that disengaged nurses are more likely to exhibit missed or delayed nursing care, reduced situational vigilance, lower levels of in‐role effort, and a form of emotional distancing that diminishes therapeutic communication with patients [21, 22]. Such behaviors typically emerge as coping responses to emotionally taxing, inequitable, or poorly led work environments. In turn, nursing‐specific withdrawal contributes to weakened team functioning, decreased care continuity, and compromised patient safety outcomes [23, 24]. Recognizing these unique manifestations is essential for understanding how destructive leadership conditions, such as exploitative leadership, may indirectly shape nurses’ clinical behaviors.

Blau’s social exchange theory (SET) [25] provides a framework for understanding the emergence of withdrawal behaviors under exploitative leadership. When nurse leaders take more than they give in the leader–follower relationship, nurses may perceive violation of reciprocal norms. This perceived imbalance may result in reduced commitment, emotional detachment, and ultimately, work withdraw behaviors. Given the relational nature of nursing, such disengagement can compromise patients’ safety and care delivery.

### 2.3. Moral Disengagement

Moral disengagement refers to a process that involves justifying one’s unethical actions by altering one’s moral perception of those actions [26]. In nursing, where ethical standards are integral to patient advocacy and clinical care, moral disengagement is particularly concerning. Research suggests that moral disengagement among nurses is associated with diminishing quality of care, increased workplace deviance, greater intention to leave the organizations, and counterproductive work behaviors [27–29]. These outcomes not only undermine professional standards but also threaten patient safety.

From a theoretical perspective, Bandura’s social cognitive theory (SCT) helps clarify how exploitative leadership can activate moral disengagement [26]. Self‐serving leaders create conditions that increase the likelihood of several disengagement mechanisms. First, moral justification may occur when leaders emphasize documentation demands or performance targets, prompting nurses to perceive missed or delayed care as unavoidable under managerial pressure [30, 31]. Second, displacement or diffusion of responsibility is facilitated by hierarchical team structures; under coercive or self‐serving leaders, nurses may attribute ethically questionable decisions to staffing shortages, workload demands, or leader directives [32, 33]. Third, distortion or minimization of consequences may arise when exploitative leaders downplay the clinical significance of fundamental nursing care activities, thereby leading nurses to perceive omissions or reduced vigilance as low‐risk events [7, 34, 35]. Finally, dehumanization may emerge when nurse leaders prioritize efficiency over person‐centered care, encouraging emotional distancing from patients and reducing compassionate engagement [36, 37].

Collectively, these mechanisms demonstrate how exploitative leadership provides the contextual cues that weaken moral self‐regulation, thereby increasing the likelihood that nurses cognitively rationalize ethically problematic behaviors. This dynamic offers a theoretically grounded explanation for why exploitative leadership predicts moral disengagement.

### 2.4. Aim

This research aims to explore the indirect effect of moral disengagement in the relationship between exploitative leadership and work withdrawal behaviors among the nursing workforce.

### 2.5. Research Hypotheses

**Figure 1:**
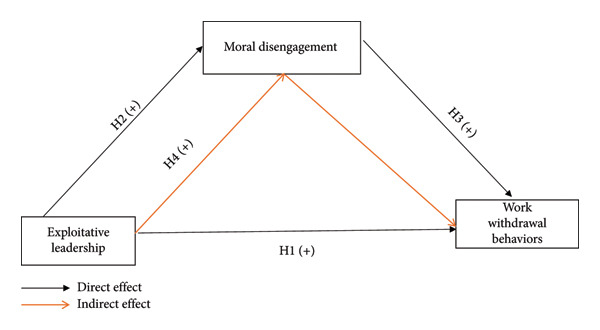
The conceptual model of the study.

This study had the following four hypotheses, which are illustrated in Figure 1:

 H1: Exploitative leadership is positively associated with work withdrawal behaviors among nurses.
 H2: Exploitative leadership is positively associated with moral disengagement among nurses.
 H3: Moral disengagement is positively associated with work withdrawal behaviors among nurses.
 H4: Moral disengagement mediates the relationship between exploitative leadership and work withdrawal behaviors among nurses.

## 3. Methods

### 3.1. Study Design

This research employed a quantitative and cross‐sectional design.

#### 3.1.1. Setting and Participants

This quantitative, cross‐sectional study included a convenience sample of 223 nurses recruited from two government‐funded healthcare settings in Al‐Kharj City, Saudi Arabia. The inclusion criteria for participants were willingness to participate, have a year of experience, active involvement in direct patient care, and hold a valid nursing license. Nurse managers, nursing interns, and professionals from other medical or paramedical disciplines were excluded.

The sample size was calculated using Soper’s software [38], yielding a minimum requirement of 184 participants based on a power of 0.95, an effect size of 0.3, a significance level of 0.05, three latent variables, and 30 observed variables. The target was established at 203 to accommodate a 10% attrition rate. A total of 250 nurses were invited to participate, yielding 223 valid and complete responses, which corresponds to a response rate of 89.2%. The remaining 27 included 15 refused and 12 ineligible participants.

### 3.2. Data Collection

Data were collected between February and April 2025 using an online survey administered via Google Forms. Prior to data collection, formal permissions were obtained from the directors of both hospitals. The aim and significance of this research were clearly demonstrated to nurse managers who facilitated survey participation. The eligible nurses were mailed with the survey link. The cover page of the Google survey included mandatory informed consent, which participants were required to accept before proceeding. The contact information for the researchers was provided to address any inquiries or concerns. To improve response rates, reminder massages were sent to nurses biweekly throughout the data collection period, encouraging them to participate. The survey remained open until an adequate sample size was reached. All survey items were set as mandatory to prevent missing data and ensure completeness of responses.

### 3.3. Measurements

#### 3.3.1. Demographic Questionnaire

This questionnaire collected data on participants’ age, gender, marital status, education, experience, and nationality.

#### 3.3.2. Exploitative Leadership Scale

Exploitative leadership scale was developed by Schmid et al. [14]; it was used to assess nurses’ perceptions of leader’s self‐serving that undermine both their interests and organizational well‐being. This scale comprises 15 items, equally distributed across five core dimensions as follows: demonstrating genuine egoistic behaviors, exerting pressure, underchallenging followers, tacking credit, and manipulating followers. The responses were rated on five‐point Likert scale range from 1 for not at all to 5 for always, with higher scores indicating greater perceived exploitative leadership behaviors.

#### 3.3.3. Moral Disengagement Scale

The 8‐item moral disengagement scale was developed by Moore et al. [39], it was used to assess the extent to which nurses cognitively justify unethical behaviors by disengaging from internal moral standards. Participants responded to each item using a five‐point Likert scale, ranging from 1 for strong disagreement to 5 for strong agreement. Higher scores indicated greater levels of moral disengagement.

#### 3.3.4. Neglect Scale

The 7‐item neglect scale was developed by Rusbult et al. [40]; it was used to capture the frequency of passive behaviors nurses engaged in to avoid work‐related duties without formally leaving the organization. The responses were rated on five‐point Likert scale range from 1 for not at all to 5 for always, with higher scores indicating greater engagement in work withdrawal behaviors.

All scales utilized in this study were openly accessible through academic databases and are not under strict copyright when used for academic purposes. The instruments were cross‐culturally adapted to Arabic language using the standardized procedures established by Beaton et al. [41] to ensure linguistics and conceptual equivalence. The adaptation process involved expert evaluation by five faculty members and two clinically experienced nurses holding master’s degrees in nursing management. These experts assessed the translated versions to establish face and content validity. Minor modifications were executed guided by the experts’ opinion as follows: the response label for option 5 on the Likert scale of exploitative leadership was revised from “frequently, if not always” to “always” to enhance clarity. In addition, the original seven‐point scales used in moral disengagement scale and neglect scale were adjusted to a five‐point format. This decision was made to maintain consistency across all instruments in the survey, thereby reducing cognitive complexity for participants and minimizing survey fatigue, which can enhance data quality.

A pilot study including 27 nurses confirmed the clarity, cultural relevance, and acceptability of the Arabic version of the scales, supporting their suitability for use in the main study. The pilot sample was not involved in the main study to prevent bias and ensure the integrity of the main study results.

### 3.4. Data Analysis

Data analysis included the examination of participants’ demographic characteristics, descriptive statistics of the study’s variables (mean scores and standard deviations), common method bias (CMB), and the evaluation of measurement model for reliability and construct validity, ensuring the appropriateness of the instruments for assessing the study’s variables. The partial least squares structural equation modeling (PLS_SEM) approach was employed using the PLS algorithm, followed by a bootstrapping procedure with 5000 subsamples to estimate the structural model and test the proposed hypotheses. Statistical significance was established at *p* < 0.05 prior to hypotheses testing, the assumptions of SEM, and potential biases. Harman’s single‐factor test indicated that the first unrotated factor accounted for 37.95% of the total variance, which is below the recommended threshold of 50%, suggesting that CMB was not a significant concern [42]. The analyses were conducted using SPSS version 27.0 and Smart‐PLs 4.1.1.2 package.

### 3.5. Ethical Considerations

This study attained ethical agreement from the standing committee of bioethics research at Prince Sattam bin Abdulaziz University, Saudi Arabia (Ref.No. SCBR‐412/2025). Participation was entirely voluntary, with informed consent obtained electronically. To ensure anonymity and confidentiality and to mitigate fears of repercussions, no personally identifiable information (e.g., name, Nurses ID) was collected at any point. The survey was administered online via a generic link, and IP addresses were not stored. The cover page of the survey explicitly stated that participation was anonymous, that their hospital management and supervisors would not have access to individual responses, and that their decision to participate or not would in no way affect their employment. All data were stored on a password‐protected computer accessible only to the research team and were used solely for the purpose of this study. The study adhered to the ethical principles of the Declaration of Helsinki.

## 4. Results

### 4.1. Characteristics of the Participants

The majority of participants (96.9%) were between the ages of 20 and 30 years, while only 3.1% were above 30 years. Most participants were females (90.1%), married (83.0%), Saudi nationals (97.3%), held a bachelor’s degree (95.1%), and had between 1 and 5 years of clinical experience (93.7%) (Table 1).

**Table 1:** Demographic information of participants.

Characteristics	*N*	%
*Age (years)*		
20–30	216	96.9
> 30	7	3.1

*Gender*		
Male	22	9.9
Female	201	90.1

*Marital status*		
Single	38	17.0
Married	185	83.0

*Nationality*		
Saudi	217	97.3
Non‐Saudi	6	2.7

*Education*		
Technical degree	11	4.9
Bachelor’s degree	212	95.1

*Experience*		
1–5	209	93.7
> 5	14	6.3

### 4.2. Assessment Measurement Model

Prior to evaluating the final measurement model, the higher‐order reflective‐reflective construct (exploitative leadership) was evaluated for validity and reliability in accordance with the guidelines of Hair and his colleagues [43]. This first assessment confirmed that the multidimensional constructs satisfied the standards for convergent and discriminant validity, in addition to internal consistency reliability (Supporting Information available here). Following this assessment, the latent scores were subsequently used in the final measurement model for further analysis.

Table 2 shows the results of the reliability and validity tests conducted on the reflective measurement model that was developed for this research. These tests included indicator loadings, discriminant validity, convergent validity, and internal consistency reliability. All indicator loading exceeded the minimum acceptable threshold of 0.708, indicating satisfactory indicator reliability. The variance inflation factor(VIF) values ranged from 1.734 to 2.630, less than the threshold of 3, suggesting the absence of multicollinearity among indicators [43].

**Table 2:** Loadings, Cronbach’s alpha, composite reliability, convergent validity, and mean scores of the constructs.

Constructs	Dimensions/items	Loading	VIF	*α*	CR	AVE	Mean (SD)
EL	DGEB	0.718	2.008	0.857	0.897	0.636	2.84 (0.65)
	EP	0.832	2.634	—	—	—	—
	UF	0.768	1.773	—	—	—	—
	TC	0.829	2.287	—	—	—	—
	MF	0.834	2.124	—	—	—	—

MD	MD1	0.807	2.272	0.918	0.932	0.634	2.51 (0.84)
	MD2	0.794	2.255	—	—	—	—
	MD3	0.847	2.630	—	—	—	—
	MD4	0.836	2.623	—	—	—	—
	MD5	0.819	2.546	—	—	—	—
	MD6	0.773	2.401	—	—	—	—
	MD7	0.771	2.311	—	—	—	—
	MD8	0.713	1.943	—	—	—	—

WWBs	WWBs1	0.749	1.879	0.887	0.912	0.596	2.41 (0.79)
	WWBs2	0.726	1.797	—	—	—	—
	WWBs3	0.796	2.103	—	—	—	—
	WWBs4	0.784	1.974	—	—	—	—
	WWBs5	0.814	2.267	—	—	—	—
	WWBs6	0.745	1.734	—	—	—	—
	WWBs7	0.787	2.151	—	—	—	—

Abbreviations: DGEB: Demonstrating Genuine Egoistic Behaviors; EL: Exploitative Leadership; EP: Exerting Pressure; MD: Moral Disengagement; MF: Manipulating Followers; TC: Taking Credit; UF: Underchallenging Followers; WWBs: Withdrawal Work Behaviors.

Internal consistency was supported by Cronbach’s alpha (*α*) values above the 0.70 benchmark for all constructs: exploitative leadership (*α* = 0.857), moral disengagement (*α* = 0.918), and work withdrawal behaviors (*α* = 0.887). Similarly, the CR values for all constructs exceeded 0.70, indicating acceptable levels of constructs reliability: 0.897 for exploitative leadership, 0.932 for moral disengagement, and 0.912 for work withdrawal behaviors. Convergent validity was also confirmed, as the AVE for each construct was above the recommended threshold of 0.50 [44], with values ranging from 0.596 to 0.636.

We used the Fornell–Larcker criteria, cross‐loadings, and the Heterotrait–Monotrait (HTMT) ratio to check for discriminant validity. Discriminant validity was further supported by HTMT values below the threshold of 0.85 [45], and square roots of AVE exceeding inter‐constructs correlations, both of which met the Fornell–Larcker criteria (Table 3). Additionally, all cross‐loadings were higher for their respective constructs than other constructs (Table 4). The mean score for exploitative leadership (mean = 2.84, SD = 0.65), suggests that participants generally perceived a moderate level of exploitative leadership within their workplace. Similarly, the mean score for moral disengagement (mean = 2.51, SD = 0.84), suggests that participants experience moderate levels of moral disengagement. In contrast, the mean score of work withdrawal behaviors (mean = 2.41, SD = 0.79), reflects a relatively low level of withdrawal behaviors among nurses (Table 2).

**Table 3:** Discriminant validity assessment.

HTMT criterion	EL	MD	WWBs
*EL*			
MD	0.646	—	—
WWBs	0.612	0.487	—

*Fornell*–*Larcker criterion*			
EL	0.797	—	—
MD	0.616	0.796	—
WWBs	0.542	0.456	0.772

Abbreviations: EL: Exploitative Leadership; MD: Moral Disengagement; WWBs: Withdrawal Work Behaviors.

**Table 4:** Cross‐loading of the items of constructs.

	EL	MD	WWBs
DGEB	**0.718**	0.362	0.365
EP	**0.832**	0.450	0.423
UF	**0.768**	0.460	0.412
TC	**0.829**	0.477	0.427
MF	**0.834**	0.643	0.507
MD1	0.646	**0.807**	0.384
MD2	0.384	**0.794**	0.401
MD3	0.655	**0.847**	0.497
MD4	0.505	**0.836**	0.312
MD5	0.444	**0.819**	0.350
MD6	0.421	**0.773**	0.344
MD7	0.402	**0.771**	0.259
MD8	0.294	**0.713**	0.264
WWBs1	0.403	0.328	**0.749**
WWBs2	0.353	0.316	**0.726**
WWBs3	0.461	0.327	**0.796**
WWBs4	0.452	0.330	**0.784**
WWBs5	0.413	0.455	**0.814**
WWBs6	0.413	0.380	**0.745**
WWBs7	0.426	0.317	**0.787**

*Note:* Bold values indicate the highest factor loading for each item, reflecting its primary association with the intended latent construct.

Abbreviations: DGEB: Demonstrating Genuine Egoistic Behaviors; EL: exploitative leadership; EP: Exerting Pressure; MD: Moral disengagement; MF: Manipulating Followers; TC: Taking Credit; UF: Underchallenging Followers; WWBs: Withdrawal work Behaviors.

### 4.3. Structural Model Assessment

The structural model was assessed to ascertain the explanatory and predictive capabilities of the constructs and the importance of the proposed correlations. The structural model was evaluated using key several metrics, including *R* ^2^ values, effect sizes (*f* ^2^), predictive relevance (*Q* ^2^), multicollinearity (VIF), and model fit indices; standardized root‐mean‐square residual (SRMR).

The *R* ^2^ value for moral disengagement was 0.38, indicating that 38.0% of the variance in moral disengagement is explained by exploitative leadership. Similarly, 31.7% of the variance in the work withdrawal behaviors was explained by exploitative leadership and moral disengagement (*R* ^2^ = 0.317). These values suggest moderate explanatory power for both endogenous constructs, consistent with the recommended thresholds by Hair and his colleagues [43]. Regarding effect sizes, exploitative leadership demonstrated a medium effect on moral disengagement (*f* ^2^ = 0.612), and small effect on both withdrawal work behaviors (*f* ^2^ = 0.161), and the effect of moral disengagement on withdrawal wok behaviors also was small (*f* ^2^ = 0.035).

The *Q* ^2^ predicted values derived through blindfolding procedures exceeded the zero threshold for both moral disengagement (*Q* ^2^ = 0.367) and withdrawal work behaviors (*Q* ^2^ = 0.280), confirming that the model holds predictive power beyond its explanatory capability [46]. The VIF values for all predictor constructs are below the critical threshold of 3, indicating no multicollinearity concerns and affirming the stability of the parameter estimates [43]. The SRMR value of 0.077 is also below the suggested threshold 0.08 [47], indicating a good model fit (Table 5).

**Table 5:** Structural model metrics.

Constructs	R‐square	R‐square adjusted	f‐square	*Q* ^2^ predict	VIF	SRMR
EL > MD	—	—	0.612	—	1.000	0.077
EL > WWBs	—	—	0.161	—	1.612	—
MD > WWBs	—	—	0.035	—	1.613	—
MD	0.380	0.377	—	0.367	—	—
WWBs	0.317	0.311	—	0.280	—	—

Abbreviations: EL: Exploitative Leadership; MD: Moral Disengagement; WWBs: Withdrawal Work Behaviors.

### 4.4. Structural Model Analysis (Hypotheses Testing)

**Figure 2:**
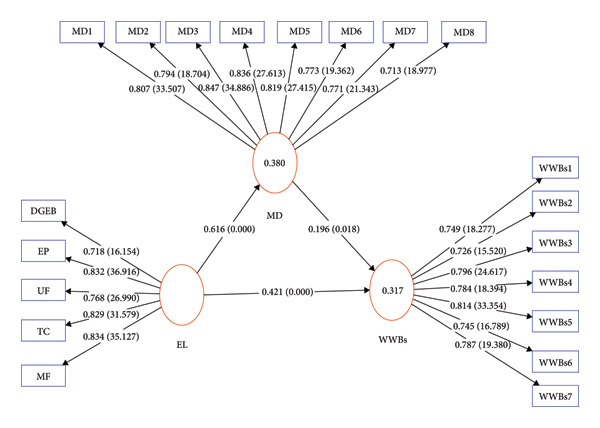
Structural model of the study. Note: Path coefficients and associated *p*‐values are presented along the direct paths. Outer loadings and corresponding *t*‐values for each indicator are displayed on the measurement model. Abbreviations: EL: Exploitative Leadership; MD: Moral Disengagement; WWBs: Withdrawal Work Behaviors.

Exploitative leadership had a significant positive direct effect on both moral disengagement (*β* = 0.616, *t* = 17.798, *p* < 0.001), and work withdrawal behaviors (*β* = 0.421, *t* = 5.592, *p* < 0.001). Additionally, moral disengagement had significant positive influence on withdrawal work behaviors (*β* = 0.196, *t* = 2.365, *p* < 0.05). The indirect effect of exploitative leadership on work withdrawal behaviors via moral disengagement was also significant (*β* = 0.121, *t* = 2.238, *p* < 0.05). The total effect of exploitative leadership on work withdrawal behaviors was substantial and significant (*β* = 0.542, *t* = 10.749, *p* < 0.001) (Table 6 and Figure 2).

**Table 6:** Bootstrapped direct, indirect, and total effects in the structural equation model.

Coefficients paths	β	T Statistics	95% CI	
			Lower	Upper
EL > MD	0.616	17.798^∗∗∗^	0.551	0.687
EL > WWBs	0.421	5.592^∗∗∗^	0.273	0.565
MD > WWBs	0.196	2.365^∗^	0.038	0.361

*Indirect effect*				
EL > MD > WWBs	0.121	2.238^∗^	0.022	0.233
Total effect	0.542	10.749^∗∗∗^	0.444	0.641

Abbreviations: EL: Exploitative Leadership; MD: Moral Disengagement; WWBs: Withdrawal Work Behaviors.

^∗^ *p* < 0.05; ^∗∗∗^ *p* < 0.001.

## 5. Discussion

This study aimed to explore the relationships among exploitative leadership, moral disengagement, and work withdrawal behaviors among nurses, with specific focus on moral disengagement as a mediating pathway.

The study demonstrated that exploitative leadership positively correlated work withdrawal behaviors among nurses, confirming H1. This finding indicates that when nurses are exposed to exploitative supervisors, those who prioritize self‐interest, manipulate others, and disregard the well‐being of subordinates are more likely to be psychologically and behaviorally disengaged from their work. This finding aligns with previous research demonstrating that destructive leadership styles, including exploitative, abusive, and toxic leadership, can lead to counterproductive work behaviors that include withdrawal behaviors, such as psychological distress, absenteeism, and disengagement in the healthcare sector especially among nurses [7, 19, 48, 49]. This finding can be explained through the lens of SET [25], the observed association may stem from perceived mistreatment and violations of ethical and relational norms by nursing supervisors, promoting nurses to reciprocate with diminished organizational commitment and increased withdrawal behaviors.

The study found that exploitative leadership positively correlated with moral disengagement among nurses, confirming H2. This outcome suggests that nurses working under exploitative leaders tend to adopt mechanisms of moral disengagement, such as rationalizing unethical behaviors, minimizing harm, or displacing responsibility to resolve the disagreement between the professional ethical standards and the unethical work environment. This finding is consistent with previous studies revealing that nurses experiencing negative affective states, such as frustration, anxiety, and powerlessness under destructive leadership conditions, are more likely to activate moral disengagement as secondary cognitive mechanism that confuses their internal moral standards, allowing them to justify ethically questionable behaviors [7, 50]. The positive correlation between self‐serving leader and moral rationalization is also grounded in Bandura’s model of moral disengagement [51]. According to this model, individuals selectively deactivate their internal moral standards to rationalize unethical or avoidant behaviors. In the context of exploitative leadership, nurses may deactivate their moral standards to justify unethical or avoidant behaviors. Exposure to repeated ethical violations can diminish moral self‐regulation, leading to nurses to use cognitive mechanisms such as moral justification or displacement of responsibility to rationalize ethical compromises without guilt. This exploitative leadership promotes climates where moral disengagement becomes a coping response.

The study revealed that moral disengagement positively influences work withdrawal behaviors confirming H3. This suggests that nurses who cognitively disengage from their moral obligations are more likely to display reduced engagement, high absenteeism, and psychological detachment from their duties. These results agreed with prior studies demonstrating that moral disengagement predicted various forms of deviant and counterproductive behaviors in workplace setting [28, 52]. Moral disengagement may enhance detachment from the emotional and ethical demands of caregiving, ultimately contributing to diminished professional conduct and quality of patient care [29]. This relationship underscores the critical role of moral cognition in shaping work‐related behaviors in healthcare settings. This finding aligns with Bandura’s model of moral disengagement of SCT, which suggests that individuals may suppress their moral standards to justify behaviors that conflict with ethical norms [51]. In nursing, such disengagement enables nurses to rationalize absenteeism or reduced effort as acceptable responses to stress or perceived injustice, ultimately diminishing self‐regulations and contributing to withdrawal from work role.

Although the association between moral disengagement and work withdrawal was statistically significant, the effect of moral disengagement on work withdrawal was small, indicating that moral disengagement explains only a modest portion of the variance in withdrawal behaviors. In applied nursing contexts, even small effects can be meaningful when considered cumulatively or in combination with other workplace stressors, particularly given the high‐stakes nature of care delivery. However, this small effect size suggests that additional determinants of work withdrawal were not captured in the current study. Prior research consistently shows that emotional exhaustion, work‐related stress, perceived organizational support, and role conflict are stronger predictors of withdrawal behaviors [53–55]. Future studies should therefore incorporate these constructs to provide a more comprehensive understanding of withdrawal dynamics among nurses and to improve the explanatory power of the model.

The research established that moral disengagement mediates the association between exploitative leadership and work withdrawal behaviors, hence confirming H4. This outcome suggests that nurses working under exploitative leaders, who may assign excessive workloads unfairly (exerting pressure) or take credit for their subordinates’ successes (taking credit), tend to adopt mechanisms of moral disengagement to cope with the cognitive dissonance between their ethical training and the unethical work environment. For example, a nurse might morally justify “cutting corners” in patient documentation by telling themselves that “everyone is overworked and it’s the only way to get through the shift” (moral justification). Alternatively, they might displace responsibility by reasoning that “if the leader doesn’t care about proper staffing, why should I kill myself trying to do everything?” (displacement of responsibility). This finding is consistent with previous nursing research revealing that negative affective states fostered by destructive leadership are linked to the activation of moral disengagement. These results reinforce SCT proposition that under adverse internal or external conditions, individuals may be cognitively disengaged from moral standards, ultimately leading to behavioral withdrawal or deviance [26].

For instance, Cheng et al. [5] found that exploitative leadership positively predicted employee expectancy through moral disengagement and Chinese traditionality moderated both direct and indirect relationships. Although their outcome variable was expectancy, a form of norm‐violating behaviors intended to serve personal goals, the underlying cognitive mechanism of moral disengagement is conceptually similar to moral rationalization that may precede work withdrawal behaviors. This evidence supports the argument that exploitative leadership diminishes moral self‐regulation, leading employees to disengage ethically and behaviorally. Moreover, Low et al. [49] revealed the central role of moral disengagement in weakening self‐regulatory processes and facilitating ethically disengaged behaviors in nursing contexts.

### 5.1. Limitations

This study has several limitations. First, this study relies on self‐reported data, which may be susceptible to social desirability bias, common method variance, and recall biases. Given the sensitive nature of topics like perceiving one’s leader as exploitative or admitting to moral disengagement, participants may have underreported their true experiences or feelings. To mitigate this, we ensured anonymity and used statistical checks (Harman’s single‐factor test), which suggested CMB was not a major concern. Nevertheless, future studies would benefit from employing multisource data collection strategies, such as pairing nurse self‐reports with supervisor ratings of withdrawal behaviors, peer evaluations of moral engagement, or objective metrics like absenteeism records, to enhance the validity and robustness of the findings. Second, the cross‐sectional nature of the data prohibits any causal inferences. While our model is grounded in theory, suggesting that exploitative leadership leads to moral disengagement and subsequent withdrawal, the reverse or bidirectional relationships are also plausible. Future research should employ longitudinal or experimental designs to track the evolution of moral disengagement over time and establish the temporal precedence of the variables, which would provide more robust evidence for causality.

Third, the generalizability of our findings is constrained by the homogeneity of the sample, which consisted predominantly of young, female, Saudi nurses from two government‐funded hospitals within a single urban area. Such demographic and cultural characteristics may systematically shape how exploitative leadership is interpreted and how withdrawal behaviors manifest. For example, hierarchical workplace norms, high power distance, and culturally embedded expectations of obedience in Saudi healthcare settings may influence both the likelihood of perceiving supervisory practices as exploitative and the range of withdrawal responses considered acceptable. These dynamics may differ markedly among older nurses, male nurses, expatriate staff, or individuals working in private‐sector institutions where organizational structures, leadership expectations, and cultural interactions are more varied.

To enhance external validity, we recommend that future studies replicate this model across more diverse regions of Saudi Arabia, other Gulf Cooperation Council (GCC) countries, and culturally distinct contexts such as Western healthcare systems. We also encourage exploration across mixed‐gender samples, varying age groups, and different employment sectors to evaluate whether the observed relationships hold under different institutional and cultural conditions. Finally, future research may examine additional psychological mechanisms (e.g., moral distress, emotional exhaustion) and contextual moderators (e.g., ethical climate, psychological safety, leadership development initiatives) to better specify the pathways and boundary conditions through which exploitative leadership influences nursing outcomes.

### 5.2. Implications of the Study

The findings have several implications for nursing management and organizational policy. First, they demonstrate the impact of exploitative leadership on nurses’ moral disengagement, underscoring the need for leadership training program that promote ethical, transparent, and supportive supervisory practices. Second, the mediating role of moral disengagement suggests that interventions aimed at strengthening moral resilience, such as ethics education, reflective practices, and peer support systems, may buffer the harmful effects of toxic leadership. From a theoretical perspective, this study advances the application of SCT in the nursing context by demonstrating how environmental stressors in the workplace can impair moral self‐regulation and foster disengaged behaviors.

## 6. Conclusions

This study sheds important light on the crucial role of moral disengagement as mediating pathway through which exploitative leadership leads to nurses’ work withdrawal behaviors. The current study revealed that exploitative leadership directly contributes to both moral disengagement and work withdrawal behaviors. Moreover, moral disengagement not only emerged as a significant outcome of exploitative leadership but also as a key mediating mechanism linking exploitative leadership to nurses’ withdrawal from their professional roles.

## Conflicts of Interest

The authors declare no conflicts of interest.

## Author Contributions

Mennat Allah G. Abou Zeid: conceptualization, data curation, funding acquisition, investigation, methodology, project administration, writing–original draft, and writing–review and editing. Amirat A. Al‐Sabeely: conceptualization, data curation, investigation, methodology, writing–original draft, and writing–review and editing. Ibrahim Abdullatif Ibrahim: conceptualization, data curation, formal analysis, investigation, methodology, project administration, supervision, validation, writing–original draft, and writing–review and editing.

## Funding

The authors extend their appreciation to Prince Sattam bin Abdulaziz University for funding this research work through the project number (PSAU/2025/03/33055).

## Supporting Information

Supporting Information. Table S1: Assessment reflective‐reflective higher‐order construct (Exploitative leadership). Table S2: Discriminant validity of the constructs. Table S3: Cross‐loading of the items of Exploitative leadership (Supporting Information).

## Supporting information


**Supporting Information** Additional supporting information can be found online in the Supporting Information section.

## Data Availability

The data that support the findings of this study are available upon request from the corresponding author. The data are not publicly available due to privacy or ethical restrictions.
