# Supplementary material for: The Impact of Exploitative Leadership and Moral Disengagement on Nurses’ Work Withdrawal Behaviors: A Mediation Model Using the Partial Least Squares Approach
Source: J Nurs Manag. 2025 Dec 31;2025:6932401. doi: 10.1155/jonm/6932401 (PMC12767590; doi:10.1155/jonm/6932401)
Supplement: Supplementary file 1 — Supporting Information Additional supporting information can be found online in the Supporting Information section. [file JONM-2025-6932401-s001.docx]

Table S1. Assessment reflective-reflective higher order construct (Exploitative leadership)

| Constructs | Dimensions | Items | Loading | VIF | Reliability | | Convergent validity |
| --- | --- | --- | --- | --- | --- | --- | --- |
|  |  |  |  |  | α | CR | AVE |
| Exploitative Leadership | DGEB | DGEB _1 | 0.838 | 1.563 | 0.784 | 0.874 | 0.698 |
|  |  | DGEB _2 | 0.818 | 1.611 |  |  |  |
|  |  | DGEB _3 | 0.849 | 1.790 |  |  |  |
|  | EP | EP1 | 0.874 | 1.881 | 0.848 | 0.908 | 0.766 |
|  |  | EP2 | 0.886 | 2.422 |  |  |  |
|  |  | EP3 | 0.866 | 2.086 |  |  |  |
|  | UF | UF1 | 0.808 | 1.535 | 0.752 | 0.858 | 0.669 |
|  |  | UF2 | 0.851 | 1.713 |  |  |  |
|  |  | UF3 | 0.793 | 1.405 |  |  |  |
|  | TC | TC1 | 0.869 | 1.879 | 0.822 | 0.894 | 0.738 |
|  |  | TC2 | 0.871 | 1.972 |  |  |  |
|  |  | TC3 | 0.836 | 1.891 |  |  |  |
|  | MF | MF1 | 0.884 | 1.726 | 0.831 | 0.899 | 0.747 |
|  |  | MF2 | 0.865 | 2.177 |  |  |  |
|  |  | MF3 | 0.845 | 1.895 |  |  |  |

*Abbreviations: DGEB: Demonstrating Genuine Egoistic Behaviors; EP: Exerting Pressure; UF: Underchallenging Followers; TC: Taking Credit; MF: Manipulating Followers.*

Table S2. Discriminant validity of the constructs

|  | DGEP | EP | UF | TC | MF |
| --- | --- | --- | --- | --- | --- |
| DGEP | **0.835** | 0.803 | 0.543 | 0.548 | 0.533 |
| EP | 0.705 | **0.875** | 0.710 | 0.644 | 0.673 |
| UF | 0.417 | 0.563 | **0.818** | 0.753 | 0.643 |
| TC | 0.444 | 0.540 | 0.592 | **0.859** | 0.832 |
| MF | 0.432 | 0.567 | 0.507 | 0.688 | **0.865** |

*Abbreviations: DGEB: Demonstrating Genuine Egoistic Behaviours; EP: Exerting Pressure; UF: Underchallenging Followers; TC: Taking Credit; MF: Manipulating Followers.*

*Note: Bolded values on the diagonals represent the square root of AVE; Lower triangle (below diagonal) represents Fornell–Larcker inter-construct correlations; Upper triangle (above diagonal) represents HTMT values (Heterotrait-Monotrait ratios).*

Table S3. Cross loading of the items of Exploitative leadership

|  | DGEP | EP | UF | TC | MF |
| --- | --- | --- | --- | --- | --- |
| DGEP1 | ***0.838*** | 0.582 | 0.366 | 0.411 | 0.375 |
| DGEP2 | ***0.818*** | 0.587 | 0.334 | 0.355 | 0.367 |
| DGEP3 | ***0.849*** | 0.598 | 0.343 | 0.341 | 0.338 |
| EP1 | 0.634 | ***0.874*** | 0.389 | 0.412 | 0.493 |
| EP2 | 0.648 | ***0.886*** | 0.466 | 0.452 | 0.456 |
| EP3 | 0.567 | ***0.866*** | 0.635 | 0.560 | 0.535 |
| UF1 | 0.351 | 0.495 | ***0.808*** | 0.492 | 0.458 |
| UF2 | 0.355 | 0.451 | ***0.851*** | 0.493 | 0.417 |
| UF3 | 0.317 | 0.435 | ***0.793*** | 0.466 | 0.370 |
| TC1 | 0.417 | 0.472 | 0.569 | ***0.869*** | 0.590 |
| TC2 | 0.423 | 0.574 | 0.509 | ***0.871*** | 0.641 |
| TC3 | 0.298 | 0.335 | 0.446 | ***0.836*** | 0.537 |
| MF1 | 0.398 | 0.510 | 0.443 | 0.702 | ***0.884*** |
| MF2 | 0.413 | 0.498 | 0.419 | 0.537 | ***0.865*** |
| MF3 | 0.307 | 0.462 | 0.454 | 0.550 | ***0.845*** |

*Abbreviations: DGEB: Demonstrating Genuine Egoistic Behaviours; EP: Exerting Pressure; UF: Underchallenging Followers; TC: Taking Credit; MF: Manipulating Followers.*
